# Coping with arsenic stress: Adaptations of arsenite‐oxidizing bacterial membrane lipids to increasing arsenic levels

**DOI:** 10.1002/mbo3.594

**Published:** 2018-03-25

**Authors:** Devanita Ghosh, Punyasloke Bhadury, Joyanto Routh

**Affiliations:** ^1^ Integrative Taxonomy and Microbial Ecology Research Group Department of Biological Sciences Indian Institute of Science Education and Research Kolkata Mohanpur West Bengal India; ^2^ Department of Thematic Studies Environmental Change Linköping University Linköping Sweden; ^3^ Present address: Laboratory of Biogeochem‐mystery Centre for Earth Sciences Indian Institute of Science Bangalore India

**Keywords:** aquifer, Arsenic, arsenite‐oxidizing bacteria, As(III) stress, phospholipid fatty acids

## Abstract

Elevated levels of arsenic (As) in aquifers of South East Asia have caused diverse health problems affecting millions of people who drink As‐rich groundwater and consume various contaminated agriculture products. The biogeochemical cycling and mobilization/immobilization of As from its mineral‐bound phase is controlled by pH, oxic/anoxic conditions, and different microbial processes. The increased As flux generated from ongoing biogeochemical processes in the subsurface in turn affects the in situ microbial communities. This study analyzes how the indigenous arsenite‐oxidizing bacteria combat As stress by various biophysical alterations and self‐adaptation mechanisms. Fifteen arsenite‐oxidizing bacterial strains were isolated and identified using a polyphasic approach. The bacterial strains isolated from these aquifers belong predominantly to arsenite‐oxidizing bacterial groups. Of these, the membrane‐bound phospholipid fatty acids (PLFA) were characterized in seven selected bacterial isolates grown at different concentrations of As(III) in the medium. One of the significant findings of this study is how the increase in external stress can induce alteration of membrane PLFAs. The change in fatty acid saturation and alteration of their steric conformation suggests alteration of membrane fluidity due to change in As‐related stress. However, different bacterial groups can have different degrees of alteration that can affect sustainability in As‐rich aquifers of the Bengal Delta Plain.

## 1 INTRODUCTION

Arsenic is found in various oxidation states such as As^5+^ (arsenate), As^3+^ (arsenite), As^0^ (elemental arsenic), and As^3−^ (arsine). Arsenic is mobilized or released into the environment by weathering or by various anthropogenic activities. This has resulted in contamination of aquifers especially in the South‐East Asian deltaic region. Millions of people living in this region particularly in the fertile Bengal Delta Plain (BDP) region are exposed to various health issues due to consumption of As contaminated groundwater and agricultural products (Pontius, Brown, & Chen, 1994). Many theories have been proposed to understand the mechanism of As release into groundwater (Bhattacharya, Chatterjee, & Jacks, 1997; Harvey et al., 2002; McArthur et al., 2004; Nickson et al., 1998), of which the microbial transformation pathway is most widely accepted. Microorganisms play an important role in the biogeochemical cycling of As and its release into the aquifer (Ghosh, Routh, & Bhadury, 2015a, 2015b, 2017; Ghosh, Routh, Dario, & Bhadury, 2015a, 2015b; Sultana, Härtig, Friedrich, Seiferta, & Schlömanna, 2011). This can occur through microbial reduction in As‐bearing Fe(III) minerals as electron sources (Bhattacharya et al., 2001; Cummings, Caccavo, Fendorf, & Rosenzweig,^1999^; Nickson, McArthur, Ravenscroft, Burgess, & Ahmed, 2000; Nickson et al., 1998; van Geen et al., 2013) or using arsenate [As(V)] as an electron acceptor (Zobrist, Dowdle, Davis, & Oremland, 2000; Oremland & Stolz, 2003; Lloyd &Oremland, 2006), after reducing Fe(III) (Islam et al., 2004). Interestingly, bacterial oxidation of the mobile As(III) species can result in immobilization of As(V). This reaction could perhaps be used as an alternate remediation method to chemical oxidation for removing As from groundwater (Rowland et al., 2007).

Several arsenite [As(III)] oxidizing bacteria have been reported to date including strains of *Achromobacter* (Green, 1918), several *Pseudomonas* spp. (Turner, 1949; Turner, 1954; Turner & Legge, 1954; Ilyaletdonov & Abdrashitova, 1981), *Alcaligenes faecalis* (Osborne and Enrlich, 1976; Philips & Taylor, 1976), *Thiobacillus ferrooxidans* and *Thiobacillus acidophilus* (Leblanc, 1995), *Herminiimonas arsenicoxydans* strain ULPAs1 (Weeger et al., 1999), bacteria from the *Agrobacterium/Rhizobium* branch of Proteobacteria(Salmassi et al., 2002; Santini, Sly, Schnagl, & Macy, 2000), bacteria of the *Thermus* genus (Gihring, Druschel, McCleskey, Hamers, & Banfield, 2001), *Thiomonas arsenivorans* strain 6 (Battaglia‐Brunet, Joulian, et al., 2006a, 2006b), *Leptothrix* sp. strain S1.1 (Battaglia‐Brunet, Itard, et al., 2006a, 2006b), *Variovorax* sp. strain4.2 (Battaglia‐Brunet, Itard, et al., 2006a, 2006b), *Achromobacter* sp. SY8 and *Pseudomonas* sp. TS44(Cai, Rensing, Li, & Wang, 2009), *Stenotrophomonas* sp. MM‐7 (Bahar, Megharaj, & Naidu, 2006) and *Geobacillus stearothermophilus* (Majumder et al.*,* 2014) that have been isolated from As contaminated environments globally. One of the main focus on isolating As(III) oxidizing bacteria is the aim to develop eco‐friendly and cost‐effective bio‐remediation methods (Bahar et al., 2006). However, the affect of As stress on such bacterial strains has been very less studied, and only change in expression of membrane protein levels in the presence of As had been shown (Podol'skaia et al.*,* 2001).

In an environmentally stressed condition, the bacterial cell wall and membrane are the first two layers of protection (Silhavy, Kahne, & Walker, 2010). Alteration of cell membrane phospholipid fatty acid (PLFA) composition to protect the cell is one of the general adaptation mechanisms in response to environmental stress (Fozo, Kajfasz, & Quivey, 2004). Such alteration in membrane‐bound PLFAs control the membrane fluidity, and is referred to as “homeoviscous adaptation” (Sinensky, 1974; Suutari & Laakso, 1974). This is achieved by the bacterial cells by modifying the degree of saturation or isomerization of *cis* to *trans* or vice versa in unsaturated fatty acids (Diefenbach, Heipieper, & Keweloh, 1992; Heipieper, Meinhardt, & Segura, 2003; Keweloh & Heipieper, 1996).

Many studies have shown these alterations in bacterial membrane composition under different environmental stress such as change in temperature, pH, presence of nutrients, and heavy metals (Denich, Beaudette, Lee, & Trevors, 2003; Fozo et al., 2004; Guerzoni, Lanciotti, & Cocconcelli, 2001; Heipieper et al., 2003; Markowicz, Płociniczak, & Piotrowska‐Seget, 2010). In the BDP aquifers, the rise in As(III) due to continuous dissolution of As‐bearing mineralsacts as a stress and plays a crucial role in shaping the microbial community structure in these regions. The predominant bacterial phylotypes belonging to *Proteobacteria* are specifically involved in the biogeochemical cycling of As in these groundwater (Ghosh, Bhadury, & Routh, 2014). To date, our knowledge about the major bacterial groups in BDP aquifers involved in various oxidative/reductive processes are mostly limited (Ghosh et al., 2014; Sultana et al., 2011). Although predominant bacterial groups in different BDP aquifers reported so far are mostly similar (Ghosh et al., 2014; Sultana et al., 2011), it remains unknown how these microbial groups adapt themselves to high As concentrations that are widely prevalent. Hence, this study aims to investigate: (1) how the increasing concentration of As(III) acts as a stress on indigenous bacteria isolated from BDP aquifers, and (2) what are the primary adaptive mechanisms exhibited by the bacterial cells (using PLFA as a tool) under increasing As(III) concentration. To the best of our knowledge this is the first study in which membrane‐bound PLFAs of indigenous bacteria isolated from the BDP aquifers have been characterized in response to increasing As(III) concentration in the cultures. The data provide an opportunity to understand complex biogeochemical interactions between bacterial communities and As cycling in these groundwater (Ghosh, Routh, & Bhadury, 2015a, 2015b, 2017).

## 2 MATERIAL AND METHOD

### 2.1 Sampling and physicochemical properties in groundwater

The diversity of As(III) oxidizing bacteria and total bacterial diversity from As‐rich groundwater collected from two different BDP aquifers was previously reported in Ghosh et al. (2014). Groundwater samples from wells 28 (N 23°55.064′, E 088°33.350′) and 204 (N 23°56.352′, E 088°33.814′) were collected, and various physicochemical properties like pH (Eco testr pH 2), temperature, conductivity, and TDS (Sartorius, PY‐Y12) were measured during sampling. To measure the concentration of dissolved elements in groundwater the samples (50 ml) were acidified by adding 2 to 3 drops of concentrated suprapure nitric acid (HNO_3_; Merck) and filtered through 0.45 μm filter. The dissolved elements were analyzed on an ICP‐MS (Perkin Elmer NexION 300D), and the detection limit of the elemental analysis was at μg/L level.

### 2.2 Bacterial count, enrichment, and isolation of As(III) oxidizing bacteria

The total bacterial cell count by DAPI staining, viable colony‐forming units (CFU) were counted by plating the groundwater samples on Luria Bertani (LB) agar plates, and incubated at 25°C for 24 hr. The total and As‐tolerant bacterial population count in the groundwater samples were determined (Bachate et al.*,* 2009). The As(III) tolerant bacteria were enriched by inoculating a chemically defined medium (CDM; Weeger et al., 1999). The CDM was prepared as follows: 100 ml solution A (consisting of 81.2 mmol/L MgSO_4_.7H_2_O, 187 mmol/L NH_4_Cl, 70 mmol/L Na_2_SO_4_, 0.574 mmol/L K_2_HPO_4_, 4.57 mmol/L CaCl_2_.2H_2_O, 446 mmol/L sodium acetate) and 2.5 ml of solution B (4.8 mmol/L Fe_2_SO_4_.7H_2_O), and 10 ml solution C (950 mmol/L NaHCO_3_) were mixed and made up to l liter with deionized distilled water. The final pH in the medium was 7.2. The media were inoculated with sample groundwater collected from the well and incubated at 25°C for 48 hr. These enriched cultures consisting of As(III) tolerant groundwater bacteria were further used for isolating the more potent As(III) oxidizing bacteria. From each groundwater sample, enrichment cultures were prepared in triplicates.

### 2.3 Isolation and screening of As(III) oxidizing bacteria

From the enrichment cultures of each well, 100 μl was plated on CDM agar (Muller et al., 2006) supplemented with 1.3 mmol/L As(III) in triplicates and incubated at 25°C for 48 hr. After incubation, from each plate of groundwater sample, 20 colonies were purified. This way, 120 colonies were purified and screened for As(III) oxidation using the silver nitrate (AgNO_3_) method (Lett, Paknikar, & Liveremont, 2001). The CDM agar plates used to purify the bacterial isolates were made in duplicates and one set was flooded with 0.1 mol/L AgNO_3_ solution. The colonies were screened based on the yellow to brown color zones formed around them. The brown color of silver orthoarsenate (Ag_3_AsO_4_) forms when AgNO_3_ reacts with As(V). Thus, colonies having brown zones were marked as As(III) oxidizers. The yellow color of silver orthoarsenite (Ag_3_AsO_3_) develops when AgNO_3_ reacts with As(III) indicating the bacterial colony to be As(III) tolerant, and not an As oxidizer. A total of 15 colonies with brown zone were selected for further screening from each groundwater sample.

### 2.4 Biochemical characterization of bacterial isolates

The oxidase activity of 30 As(III) oxidizing bacterial isolates was determined by oxidation of 1% p‐amino dimethylaniline oxalate and their catalase activity was determined with 3% (v/v) H_2_O_2_ solution. To determine the carbon substrate utilization pattern in isolates 30 different organic compounds were used as sole carbon source; the tests were performed with Carb Kit (HiMedia) following the manufacturer's instructions.

### 2.5 Heavy metals tolerance of bacterial isolates

The identified bacterial strains were also tested for their tolerance to other trace elements (e.g., Cu, Cd, Cr, Ni, and Hg). The As(III) oxidizing bacterial isolates were grown in series of LB media and supplemented with salts with concentrations (0 to 20 mmol/L); the concentrations were increased in the order of 1 mM of trace metals: cadmium (Cd), cobalt (Co), chromium (Cr), copper (Cu), nickel (Ni), and mercury (Hg), The tolerance to As(V) was tested by growing the isolates in series of LB media supplemented with sodium arsenate; the concentration was increased by 10 mmol/L up to a final concentration of 300 mmol/L. The tubes were incubated at 25°C for 48 hr and the minimum inhibitory concentration (MIC) of each metal, beyond which no growth was observed recorded as part of the protocol.

### 2.6 16S rRNA and *aioA* gene amplification

Of the 30 bacterial isolates, 15 strains showed significant As(III) oxidizing capability (Table 3) and could grow in media supplemented with As(III) concentration above 15 mmol/L. These strains were selected for downstream molecular analyses. The genomic DNA of bacterial isolates were extracted (Sambrook & Russel, 2001) and PCR amplification of the bacterial 16S rRNA fragments were targeted using previously published eubacterial primers Fc27 (5′‐AGAGTTTGATCCTGGCTCAG‐3′) and Rc1492 (5′‐TACGGCTACCTTGTTACGACTT‐3′) (Lane, 1991) Each PCR reaction consisted of 0.5 μl DNA Dream *Taq* polymerase (5 U/μl) (Fermentas), 5.0 μl 10x Dream *Taq* buffer, 5.0 μl dNTPs (final concentration 0.2 mmol/L), 5.0 μl MgCl_2_ (final concentration 2.0 mmol/L), 0.5 μl of each primers (final concentration 5 μmol/L), 0.5 μl (~ 20 ng) DNA template, 0.5 μl BSA (1 mg/ml), and nuclease‐free water to make a final volume of 50 μl. The PCR conditions applied were as follows: initial denaturation at 95°C for 10 min, 36 cycles of 95°C for 1 min, 55°C for 1 min, 72°C for 3 min 30 s, and final extension at 72°C for 10 min. The PCR reactions were performed in triplicates, and subsequently pooled down and gel purified using the Gel Purification Kit (Qiagen) as per the manufacturer's instruction.

To detect the presence of arsenite oxidase enzyme, and to determine the functional gene‐based phylogeny of the 15 selected bacterial isolates, the larger subunit AioA of the enzyme arsenite oxidase was targeted. Amplification of partial fragment of the larger subunit of the *aioA* gene fragments were undertaken from genomic DNA extracted from the isolated bacteria using the earlier reported primers *aioAF* (5′CCACTTCTGCATCGTGGG 3′) and *aioAR* (5′ TGTCGTTGCCCCAGATGA 3′) (Ghosh et al., 2014). Each PCR reaction contained 0.5 μl (~20 ng) of DNA template, 5.0 μl 10X Dream *Taq* buffer, 5.0 μl dNTPs (final concentration 0.2 mmol/L), 5.0 μl MgCl_2_ (final concentration 2.0 mmol/L), 0.5 μl each of the *aioA* primers (final concentration 5 μmol/L), 0.5 μL BSA (1 mg/ml), 0.5 μl DNA Dream *Taq* polymerase (5 U/μl) (Fermentas), and nuclease‐free water to make a final volume of 50 μl. The PCR conditions were as follows: initial denaturation at 95°C for 10 min, 35 cycles of 95°C for 1 min, 70°C for 1 min, 72°C for 2 min 30 sec, and final extension at 72°C for 10 min. The size of *aioA* amplicons were approximately 1114 bp.

All the PCR amplicons (16S rRNA and *aioA* fragments) were purified using the Gel Purification Kit (Qiagen) as per the manufacturer's instructions, and sequenced in both directions using respective primers in an ABI Prism 3730 Genetic Analyzer based on Big Dye Terminator chemistry.

### 2.7 Phylogenetic characterization of bacterial isolates

The chromatograms of the sequences were checked manually for miss‐spaced peaks, peak shifts, and double peaks using Bio Edit (version 7.1.3; Hall, 1999). The 16S rRNA sequences were checked in Bellerophon (Huber, Faulkner, & Hugenholtz,^2004^) for chimera, and were compared in nucleotide databases (GenBank/EMBL/DDBJ). The 16S rRNA sequences were aligned with the earlier published 16S rRNA sequences having the closest identity, and a phylogenetic tree was constructed based on the maximum likelihood (ML) method (Saitou & Nei, 1987) and Kimura 2 parameter (Kimura, 1980). To root the tree, the 16S rRNA sequence of *Bacillus subtilis* BCRC 10058 (Acc.No. DQ993674) was used as an outgroup.

For the *aioA* nucleotide sequences in the BDP, As(III) oxidizing bacterial isolates were translated into amino acid sequences using EMBOSS Transeq (Rice, Longden, & Bleasby, 2000), and were compared against the protein databases (GenBank/EMBL/PDB) using the blastp tool (Camacho et al., 2008). The *aioA* amino acid sequences were aligned with earlier published *aioA* sequences that have closest identity and a phylogenetic tree was constructed based on the NJ method on JTT model (Jones, Taylor, & Thornton, 1992). The tree was rooted using the *aioA* gene sequence of *Thermus aquaticus* Y51MC23 (Acc.No. EED09253).

### 2.8 PLFA characterization of selected bacterial isolates

The seven selected bacterial isolates from which *aioA* gene could be amplified, were subsequently grown in the LB media supplemented with different concentrations of As(III) (1 mmol/L, 2 mmol/L, 5 mmol/L, and 10 mmol/L As). When the optical density (OD) of bacterial culture reached 1, the cell pellet was harvested by centrifuging at 3,000 *g* for 15 mins. The pellet was washed twice with normal saline water (0.9% NaCl in water) and freeze‐dried. Total fatty acid was extracted modifying the earlier published protocol (Lores, Gómez‐Brandón, Pérez‐Díaz, & Domínguez, 2006). Approximately, 50 mg of the freeze‐dried bacterial pellet was mixed with 30 ml of chloroform–methanol mixture (2:1v/v) in 100 ml sterilized and combusted glass vials. The vials were shaken vigorously for 30 min, and the mixture separated at room temperature for 24 hr. The supernatant was collected in a glass test tube and then evaporated to dryness under a gentle stream of oxygen‐free N_2_ gas. The total lipid extract obtained was dissolved in chloroform (3 × 1 ml) and fractionated into neutral lipids, glycolipids, and phospholipids, with chloroform (5 ml), acetone (10 ml) and methanol (5 ml) by solid phase extraction using amino‐propyl cartridges (500 mg/6 ml). Phospholipid extracts were re‐dissolved in 1 ml of methanolic‐boron trifluoride (BF_3_), and heated at 100°C for 2 hr. The samples were dried and 3 ml of 5% NaCl solution and 2 ml of hexane were added, mixed vigorously and centrifuged at 1,000 *g* for 10 min to separate the layers. The hexane layer was separated, and the extraction was repeated three times. A pinch of sodium sulfate (Na_2_SO_4_) was added and left overnight at room temperature to absorb moisture. The hexane extract removed and reduced to ~1 ml by roto‐evaporation. The internal standard deuterated eicosanoic acid methyl ester (d‐EAME) spike of 10 mg/l was added, and the samples were analyzed by gas chromatography‐mass spectrometry (GC‐MS) approach.

### 2.9 Gas chromatography–mass spectrometry analyses

The PLFA samples were analyzed in an Agilent 6890N GC interfaced to an Agilent 5973 MSD mass spectrometer at 70 eV and scanned from *m/z* 40–600 at 2.62 scans/s. The samples were injected in split‐less mode (1 μl; inlet pressure of 10 psi with a flow rate 54.3 ml/min), and separated on a HP‐5 MS capillary column (5% diphenyl dimethyl polysiloxane; length 30 m, 250 μm, film thickness 0.25 μm). A constant flow (1.3 ml/min) of He was used as carrier gas. The temperature of interface was set at 300°C; and the mass source temperature was set at 230°C and the MS quadrupole was maintained at 150°C, respectively. The samples were injected at 35°C and the oven was programmed to 130°C at 20°C/min and then at 6°C/min to 320°C where it was maintained isothermally for 15 min. Based on the retention time and mass spectra of different lipids, the compounds were identified by comparing with the NIST library, and individual fatty acid standard mix from Sigma‐Aldrich. The PLFAs were quantified with respect to the response of the internal standard.

### 2.10 Change in PLFA profile with As(III) concentration

To determine the effect of As toxicity on bacterial cell membrane, the selected bacterial isolates were grown in LB media supplemented with four different concentrations of As(III), that is, 1 mmol/L, 2 mmol/L, 5 mmol/L and 10 mmol/L. Once the optical density of the cultures was 1, the cells were pelleted down and the PLFA was extracted using above mentioned method, and analyzed in a GC‐MS (as detailed above).

## 3 RESULTS

### 3.1 Physicochemical properties and bacterial isolation

The physicochemical properties and dissolved elemental profile of groundwater samples are detailed in Table 1. The concentration of As, Fe, Mn, Mg, and P were higher in groundwater from well 204 compared to well 28. The total bacterial cell counts in groundwater samples were 36,000 cells/ml in well 28 and 28,000 cells/ml in well 204. The viable cell count was 127 cells/ml and 107 cell/ml in well 28 and 204, respectively. Enrichment of As(III) oxidizing bacteria was done in CDM supplemented with 1.3 mmol/L As(III), and bacterial isolates were screened and selected based on AgNO_3_ test having brown zones around them. The biochemical and carbon substrate assimilation test of the 30 selected bacterial isolates were carried out, and the results are detailed in Table S1.

**Table 1:** Physicochemical properties of groundwater

Sampling site	Dissolved elemental concentrations in groundwater											Physicochemical parameter of groundwater				
	As^a^	Fe^a^	Mn^a^	Mo^a^	Mg^a^	Si^a^	P^a^	S^a^	Li^a^	K^a^	Na^a^	pH	Water Temp (°C)	Air Temp (°C)	Ionic Conductivity (μS/cm)	TDS
Well 28	0.055	1.65	0.30	0.002	14.81	6.35	0.06	0.47	0.002	1.52	8.778	7.3	22.3	16.5	717	358
Well 204	0.11	3.45	0.48	0.002	19.64	6.23	0.12	0.41	0.002	1.45	10.51	7.3	23.4	17.2	758	379

a in mg/l.

To test the As(III) tolerance capacity of the 30 screened As(III) oxidizing bacterial isolates, they were grown in media supplemented with different concentrations of As(III) along with *E. coli* DH5α and *B. subtilis* as control cultures (Table 2). Based on the wide range of reports on bacterial tolerance capacity to As(III) in the media (Ghosh et al., 2014; Heinrich‐Salmeron et al., 2011; Katsoyiannis & Zouboulis, 2006; Sultana et al., 2011), it was found that the common bacterial isolates could tolerate up to 15 mmol/L, many strains could tolerate up to 17 mmol/L but there were very few strains that could tolerate up to 20 mmol/L As(III). Hence, the bacterial isolates in this study were grouped as: low arsenite tolerant (LAT), medium arsenite tolerant (MAT), and high arsenite tolerant (HAT) strains. The LAT were isolates that could grow in As(III) concentration ranging from 7 to 14 mmol/L, MAT could grow in As(III) concentration ranging from 15 to 17 mmol/L, and HAT could grow in As(III) concentration ranging from 18 to 20 mmol/L. The MIC for other trace metals in the cultures for these isolates was also determined (Table 2). The isolates were regrouped as LAT, MAT, and HAT based on their tolerance to As(III) following the similar pattern of grouping for other trace metals evaluated as part of this study. Of the 30 As(III) oxidizing bacterial isolates, 15 belonged to LAT, 9 belonged to MAT and 6 belonged to HAT. The 15 MAT and HAT bacterial isolates were selected for further analyses.

**Table 2:** Minimum inhibitory concentration (MIC) for different elements inhibiting growth of each bacterial isolated strain

Elemental concentration in mM	Bacterial strain name																													
	BDP 1	BDP 2	BDP 3	BDP 4	BDP 5	BDP 6	BDP 7	BDP 8	BDP 9	BDP 10	BDP 11	BDP 12	BDP 13	BDP 14	BDP 15	BDP 16	BDP 17	BDP 18	BDP 19	BDP 20	BDP 21	BDP 22	BDP 23	BDP 24	BDP 25	BDP 26	BDP 27	BDP 28	BDP 29	BDP 30
**As(III)**	16	17	16	16	10	10	12	7	9	16	15	16	16	12	20	12	12	20	20	20	16	9	20	18	10	11	11	7	11	12
**As(V)**	250	260	260	260	170	160	160	110	130	250	210	210	220	180	260	180	170	260	260	290	230	120	280	280	130	110	110	90	120	130
**Cd**	14	14	12	14	14	12	14	2	14	14	14	15	15	12	15	12	12	15	15	15	14	14	15	16	12	10	10	2	12	14
**Hg**	0	0	0	0	0	0	0	0	0	0	0	0	0	0	0	0	0	0	0	0	0	0	0	0	0	0	0	0	0	0
**Cr**	6	6	6	6	6	5	6	0	4	6	6	6	6	4	8	4	2	5	6	3	6	4	7	6	8	4	4	0	5	6
**Ni**	8	7	6	7	4	4	5	0	4	5	6	7	8	4	6	4	4	6	6	2	5	4	8	7	6	2	2	0	4	5
**Cu**	2	2	1	1	0	<1	0	0	0	0	0	2	2	2	0	2	0	2	0	2	0	0	2	2	2	0	0	0	0	0
**Co**	7	8	7	7	10	7	6	<1	5	5	6	7	8	7	7	4	<1	7	7	7	7	4	7	7	7	5	5	<1	5	6

No color indicates LAT; light gray indicates MAT; deep gray indicates HAT.

### 3.2 Molecular phylogeny

**Figure 1:**
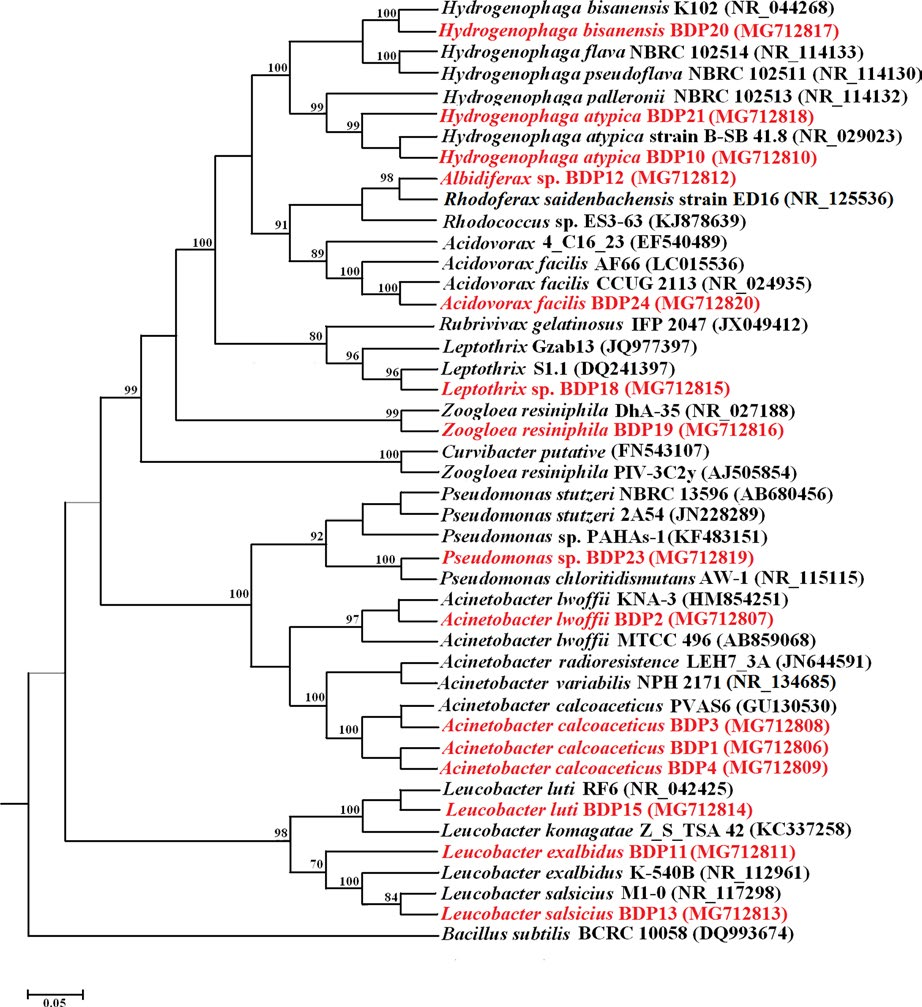
Maximum likelihood phylogenetic tree of 16S rRNA gene sequences of the bacterial isolates (red‐this study).The 16S rRNA gene of *Bacillus subtilis* BCRC 10058 (Acc. No. DQ993674) was used as outgroup to root the tree. [Scale bar indicates 0.05 substitutions per site; bootstrap approach implemented based on 1,000 replicates of original data set, only bootstrap values above 50% are shown]

The 16SrRNA sequences of the isolates showed that most of the strains belong to the bacterial phylum *Proteobacteria*, whereas a few belong to the phylum *Actinobacteria* (Figure 1). Under *Proteobacteria,* two major bacterial classes were detected namely *Betaproteobacteria* and *Gammaproteobacteria*. Under the phylum *Proteobacteria*, the isolates BDP10, BDP12, BDP18, BDP19, BDP20, BDP21, BDP23, and BDP24 belong to the order *Betaproteobacteria* and the isolates BDP13 and BDP15 belong to the order *Gammaproteobacteria*. The isolates BDP1, BDP2, BDP3, and BDP4 belonged to phylum *Actinobacteria,* class *Actinobacteria* (Figure 1). The16S rRNA sequences from the BDP bacterial isolates have been submitted to GenBank and their closest identity at the nucleotide level with published cultured bacterial 16S rRNA sequences available in the GenBank/RDB/EMBL/DDBJ databases are further detailed in Supplementary Table S2. Additionally, the GC% of 16S rRNA for each bacterial isolate has been detailed in Table S2.

**Figure 2:**
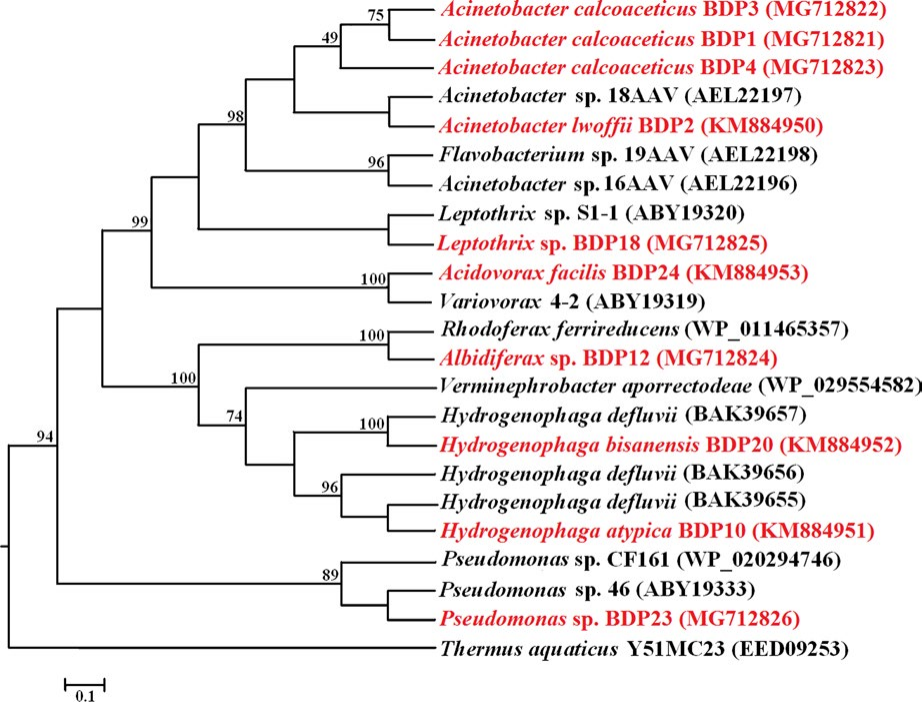
Maximum likelihood phylogenetic tree of amino acid sequences of aioA gene of the bacterial isolates (red‐this study). The amino acid sequences of aioA gene of *Thermus aquaticus* Y51MC23 (Acc. No. EED09253) was used as outgroup to root the tree. [Scale bar indicates 0.1 substitutions per site; bootstrap approach implemented based on 1,000 replicates of original data set, only bootstrap values above 50% are shown]

To check if the 15 bacterial isolates were obligate As(III) oxidizer and not merely As(III) tolerant, the *aioA* gene was amplified and sequenced. Out of these 15 isolates, 10 isolates (BDP1, BDP2, BDP3, BDP4, BDP10, BDP12, BDP18, BDP20, BDP23, and BDP24) indicated positive amplification for this gene. For validation of the newly designed primers, the bacterial strains *A. lwoffii* strain BDP2 (Acc.No.KM884950), *H. atypica* strain BDP10 (Acc.No.KM884951), *H. bisanensis* strain BDP20 (Acc.No.KM884952), and *A. facilis* strain BDP24 (Acc.No.KM884954) were used earlier (Ghosh et al., 2014). The phylogeny of the bacterial isolates based on their functional gene *aioA* was assessed (Figure 2). All the obligate As(III) oxidizers belonged to the bacterial phylum *Proteobacteria*. The *aioA* gene sequence at the amino acid level from the BDP bacterial isolates showed closest sequence identity with published cultured bacterial *aioA* sequences available in GenBank/EMBL/DDBJ/PDB databases and are further detailed in Supplementary Table S3.

### 3.3 Phospholipid composition

Since extraction, purification, characterization, and quantification of membrane phospholipid is a laborious process, of the 10 arsenite‐oxidizing bacterial isolates, which were identified by their 16S rRNA and functional gene *aioA* sequences, only seven strains were further selected based on their generic similarities. The predominant membrane phospholipid content of all seven bacterial isolates grown in the control media consisted of C14:0, C16:0, C16:1, C18:0, and C18:1. These four monomers consisted of about 70% of whole‐membrane phospholipid extract (Supplementary Table S4). In *Acinetobacter lwoffi* strain BDP2 the major cellular fatty acids were as follows: C10:0, C12:0, C14:0, C15:0, C16:0, C18:0, C20:0, Ci14:0, Ci15:0, Ca15:0, Ci16:0, Ci17:0, and Ca17:0 (Fig. S1a). In *Hydrogenophaga atypica* strainBDP10 the major cellular fatty acids were as follows: C10:0, C12:0, C14:0, C15:0, C16:0, C18:0, C20:0, Ci14:0, Ci15:0, Ca15:0. In *Albidiferax* sp. strain BDP12 the major cellular fatty acids were as follows: C10:0, C12:0, C14:0, C15:0, C15:0, C16:0, C18:0, Ci14:0, Ci15:0, Ca15:0, Ci16:0, Ci17:0, and Ca17:0. In *Leptothrix* sp. strain BDP18 the major cellular fatty acids were as follows: Ci14:0, Ci15:0, Ca15:0, Ci16:0, Ci18:0, Ci14:0, Ci15:0, Ci16:0, Ci17:0, and Ca17:0. In *Hydrogenophaga bisanensis* strain BDP20 the major cellular fatty acids were: C14:0, C15:0, C16:0, C17:0, and C18:0. In *Pseudomonas* sp. strain BDP23 the major cellular fatty acids were as follows:C14:0, C16:0, and C18:0. In *Acidivorax facilis* strain BDP24 the major cellular fatty acids were as follows:C14:0, C16:0, C18:0, C18:1ω9t, Ci15:0, Ca15:0, and Ci16:0.

Earlier studies had shown that alteration in bacterial membrane fatty acid composition may lead to decrease in the proportion of monounsaturated fatty acids (Fozo & Quivey, 2004; Quivey, Faustoferri, Monahan, & Marquis, 2000). One of such studies suggested a ratio (named here as FQ ratio; Fozo & Quivey, 2004) to detect the relative saturation extent:

$$\text{FQ Ratio}=\frac{\left(C14:0+C16:0\right)}{\left(C18:1+C20:1\right)}$$

However in a similar study it had been shown that as a result of heavy metal stress the degree of saturation in odd chain fatty acids increase (Markowicz et al., 2010). Because the C15 and C17 unsaturated and saturated fatty acids were the most abundant odd‐chain fatty acids, these monomers were selected to derive a new ratio “R” which was used to detect such alterations in bacterial PLFAs where:

$$R=\frac{\left(C15:0+C17:0\right)}{\left(C15:1+C17:1\right)}$$

Alteration in *cis*‐*trans* isomerization in the isolates was assessed based on the ratio (I),

$$I=\frac{C18:1\omega 9t+C18:2\omega 6}{\left(C14:1\omega 5c+C15:1\omega 5c+C16:1\omega 7c+C17:1\omega 7c+C18:1\omega 9c+C18:2\omega 6c+C18:3\omega 6c+C18:3\omega 3c\right)}$$

The ratios (FQ, R, and I) for PLFA extracted from all seven bacterial isolates grown in different concentrations of As(III) are detailed in Table 3. In *Acinetobacter lwoffi* strain BDP2, with increase in As(III) concentration in growth media, the total PLFA concentration decreased. The FQ ratio did not show much difference. This was clear when the fatty acid ratio R decreased with increase in PLFA saturation in the growth media. The ratio I also showed some relation with As(III) concentration, but this trend was more specific only in case of certain bacteria. In *Hydrogenophaga atypica* strain BDP10 with increase in As(III) levels in the growth media, the total PLFA concentration reduced and the R and I ratios decreased sharply indicating increase in PLFA saturation, and also *trans* isomerization. Similar changes were observed in *Leptothrix* sp. strain BDP18, *Hydrogenophaga bisanensis* strain BDP20, *Pseudomonas* sp. strain BDP23 and *Acidivorax facilis* strain BDP24. However, in *Albidiferax* sp. strain BDP12 the ratio “I” did not correlate well with increase in As(III) concentration in the growth media. Overall, the FQ ratio did not show a good relationship with As(III) concentration.

**Table 3:** Ratios used to study alteration in membrane fatty acid composition of bacterial isolates

Isolate Name	R^a^					I^b^				
	0 mmol/L	1 mmol/L	2 mmol/L	5 mmol/L	10 mmol/L	0 mmol/L	1 mmol/L	2 mmol/L	5 mmol/L	10 mmol/L
BDP2	7.129	2.121	1.901	1.846	0.002	0.149	0.201	0.346	0.236	0.104
BDP10	13.545	3.882	2.220	1.213	0.004	4.543	1.018	0.683	0.696	0.852
BDP12	9.567	5.230	4.265	3.005	0.002	7.69	1.32	3.45	3.41	1.06
BDP18	7.735	2.516	1.958	1.810	0.002	7.25	1.58	1.07	0.78	0.87
BDP20	8.521	5.917	3.752	0.003	0.025	11.68	1.41	2.29	0.82	0.48
BDP23	1.939	1.967	1.652	0.012	0.004	5.45	12.79	2.79	1.60	0.59
BDP24	12.378	7.912	1.365	1.154	0.070	3.82	0.47	0.60	1.08	1.21

a R= Unsaturated fatty acid/Saturated fatty acid.

b I = Cis fatty acids/Trans fatty acids.

## 4 DISCUSSION

The remobilization of As in the BDP groundwater is influenced by chemical and microbial oxidation‐reduction processes in different ecological niches in the aquifers. The increase in As(III) level in groundwater puts a counter stress on the indigenous microbial communities affecting their distribution. Microbial population was studied in two different aquifers of this region. The total bacterial cell count and viable cell counts are relatively close in both the aquifers. Overall, the strains isolated from those aquifers had a very high MIC for As(III) in comparison to mesophilic bacteria like *E. coli* DH5α and *B. subtilis*. It was observed that the isolated strains that had a higher MIC for As(III), also had higher MIC for other trace metals too. The phylogenetic identification of highly tolerant bacteria showed close taxonomic affiliation to the dominant arsenite‐oxidizing bacterial 16S rRNA and *aioA* sequences reported earlier from the BDP aquifers (Ghosh et al., 2014). This indicates congruence between the uncultured‐ and culture‐dependent approaches. The Burkholderial genera *Acidovorax* and *Hydrogenophaga* indicated predominance over all the arsenite‐oxidizing groups in many different BDP aquifers (Ghosh et al., 2014, 2017; Ghosh, Routh, & Bhadury, 2015a, 2015b; Ghosh, Routh, Dario, et al., 2015a, 2015b); Heinrich‐Salmeron et al., 2011; Sultana et al., 2011). These two bacteria participate in oxidation/reduction reactions involved in Fe cycling colinked to As biogeochemical cycling (Ghosh et al., 2014; Meyer‐Dombard, Amend, & Osburn,^2012^). Moreover, the isolate *Leptothrix* sp. strain BDP18 belong to one of the well‐known freshwater iron‐oxidizing bacterial genus that can coprecipitate As(III) as As(V), and also detoxify it (Katsoyiannis & Zouboulis, 2006). On the other hand, the isolate *Albidiferax* sp. strain BDP12 belong to an iron reducing bacterial genus (Finneran et al.*,* 2003), which take part in reductive dissolution of arseniferous iron‐oxy‐hydroxide resulting in dissolution of Fe and As into the BDP groundwater (Ghosh et al., 2014, 2017; Ghosh, Routh, & Bhadury, 2015a, 2015b; Ghosh, Routh, Dario, et al., 2015a, 2015b).

Various studies have shown that bacterial cells develop a wide range of adaptive mechanisms in response to external stress applied to the cell membrane (Milisav, 2011; Zhang & Rock, 2008). This may induce cellular auto‐repair options or autolysis (Perry, Jones, Peterson, Cvitkovitch, & Levesque, 2009). In this study, where membrane PLFAs were used as a proxy to study such adaptive mechanisms, the selected bacterial strains respond differently to the As(III) concentrations and their respective PLFA characteristics. An overall decrease in total PLFA concentration occurs with increase in As(III) concentration. The decrease could be due to improper synthesis of membrane lipids in the presence of As(III) (Zhang & Rock, 2008). Moreover, increase in cell death before harvesting the cell mass leading to PLFA degradation (Kunihiro et al., 2014) cannot be ruled out.

Earlier studies show that alteration in bacterial membrane fatty acid composition may lead to decrease in the proportion of monounsaturated fatty acids (Fozo & Quivey, 2004; Quivey et al., 2000). The FQ Ratio (Fozo & Quivey, 2004) used in earlier studies to detect the relative saturation in fatty acids, only considers the even‐chain fatty acids. However, a recent study shows that stress induced by exposure to trace elements on bacteria, the degree of saturation in odd‐chain fatty acids also increase (Markowicz et al., 2010). Because the C_15_ and C_17_ unsaturated and saturated odd‐chain fatty acids are the most abundant monomers, they were selected to derive a new ratio “R.” The R value decreases significantly with increase in As(III) stress in all the bacterial strains (Table 3) indicating that the degree of saturation is most abundant in fatty acids. Such alteration in saturation occurs in both even and odd‐chain fatty acids. The change results in decrease in membrane fluidity and permeability that makes the membrane more rigid and stable reducing the proton channels, and other transporter proteins binding the membrane (Dufourc, Smith, & Jarrell, 1984; Dunkley, Guffanti, Clejan, & Krulwich, 1991). The high degree of unsaturation in membrane lipids makes it more susceptible to free radical attack (García, 2005). The increase in saturation of bacterial membrane fatty acids perhaps represents the bacterial cell defense aimed at reducing the damage caused by oxidative radicals generated by trace metals (Howlett & Avery, 1997).

Other than alteration in membrane PLFA saturation, several studies have shown that *cis‐trans* isomerization of fatty acid monomers is one of main response to stress in bacterial cells (Denich et al., 2003; Heipieper et al., 2003). Consistent with this, the “I” value decreases with increase in As(III) stress in all bacterial strains indicating an increase in *cis‐trans* isomerization. This change occurs in all strains except *Acinetobacter lwoffi* strain BDP2 and *Albidiferax* sp. strain BDP12 (Table 3). The alteration in steric conformation in the bacterial membrane structure also leads to decrease in membrane fluidity (Heipieper et al., 2003; Seelig & Waespe‐Šarcevic, 1978). Similar adaptive mechanisms with alteration in membrane fatty acid composition of bacterial cells in response to external stressors are reported in *Vibrio* and *Pseudomonas* spp.(Heipieper et al., 2003; von Wallbrunn, Richnow, Neumann, Meinhardt, & Heipieper, 2003). The trans‐fatty acids are formed by direct isomerization of the complementary cis‐configuration of the double bond without any shift in its position (von Wallbrunn et al., 2003). Similar changes in steric conformation is absent in *Acinetobacter lwoffi* strain BDP2 and *Albidiferax* sp. strain BDP12. The exact reason remains unknown, but nonalteration/reverse alteration in membrane lipids to conserve the bilayer structure has been suggested in the aerobic bacterium *Acinetobacter* (Kabelitz, Santos, & Heipieper, 2003) as a possible mechanism.

It is evident that high As level acts as a stressor to obligate As metabolizing species, and this change triggers a response to adjust to the stress. Increase in As levels in the BDP groundwater increases the stress on microbial communities, resulting in a natural selection pressure generated within the community to offset the high stress level. Only those bacterial groups that are able to adapt invoking various metabolic alteration, can sustain under prevalant stressed conditions. This results in predominance of certain bacterial groups and exclusion of others in these aquifers. Furthermore, the change triggers the development of microbial niche that helps these bacteria to survive under environmental stress of high As levels in groundwater. Thus, As stress in these aquifers plays a major role in shaping the microbial community structure, as revealed by earlier study (Ghosh et al., 2014).

## 5 CONCLUSIONS

It had been largely reported that a contaminant can affect the microbial population structure of an ecosystem as in sediments or groundwater based on the change in total PLFA content. The effect of any contaminant on individual bacterial cells has never been studied. The BDP aquifers have largely been reported to be contaminated with high levels of As. The different indigenous microbial groups play a major role in the cycling of this contaminant in groundwater. In this study, we isolated bacterial strains that belong to these predominant groups, and used their membrane PLFAs as a tool to study the effect of As(III) stress. A large number of bacterial strains were isolated and their Gram character, biochemical characterization for phenotypic identification and carbohydrate utilization pattern were tested. Out of these, a few were selected based on their MIC for As. and they were further identified using multiple approaches including sequencing of 16S rRNA, *aioA* gene, and PLFA characterization. Based on their identity and phylogenetic affiliation at the generic level, seven bacterial isolates were selected for further analyses. These isolates were grown in the presence of different concentrations of As(III) in media, and the stressed bacterial cells were harvested and their PLFAs characterized. Although low dose of As(III) can trigger a stress response in these cells involving alteration in their bacterial PLFA characteristics, the cut‐off level that triggers the response differs from one genus to another. Overall, the study shows the potential for using PLFA analysis in studying bacterial stress physiology to study elemental contamination in natural ecosystems.

## ACKNOWLEDGMENTS

Help extended by Susanne Karlsson and Lena Lundman in the laboratory is appreciated. We are thankful to Bo Svensson for his encouragement and ideas. We also thank the reviewers for their suggestions and comments. Funding for this study was provided by the Swedish Research Link Asia Program (Grant2009‐6470). This work is partly supported by IISER Kolkata grant awarded to PB. DG thanks the Department of Science and Technology, Government of India for providing the INSPIRE fellowship.

## CONFLICT OF INTEREST

The authors declare no conflicts of interest.
