# Supplementary material for: Coping with arsenic stress: Adaptations of arsenite‐oxidizing bacterial membrane lipids to increasing arsenic levels
Source: Microbiologyopen. 2018 Mar 25;7(5):e00594. doi: 10.1002/mbo3.594 (PMC6182550; doi:10.1002/mbo3.594)
Supplement: Supplementary file 1 [file MBO3-7-e00594-s001.docx]

**Supplementary tables**
Table S1. Biochemical characterization of all isolated bacterial strains.

| **Strain Name** |  | Catalase activity | Oxidase activity | Starch hydrolase activity |  | Nitrate reductase activity | **Carbohydrate utilization** | | | | | | | | | | | | | | | | | | | | | | | | | | | | | | | | | |
| --- | --- | --- | --- | --- | --- | --- | --- | --- | --- | --- | --- | --- | --- | --- | --- | --- | --- | --- | --- | --- | --- | --- | --- | --- | --- | --- | --- | --- | --- | --- | --- | --- | --- | --- | --- | --- | --- | --- | --- | --- |
|  | Gram Charater |  |  |  | Esculin hydrolysis |  | Lactose | Xylose | Maltose | Fructose | Dextrose | Galactose | Raffinose | Trehalose | Melibiose | Sucrose | L-Arabinose | Mannose | Innulin | Sodium Gluconate | Glycerol | Salicin | Dulcitol | Inositol | Sorbitol | Mannitol | Adonitol | Arabitol | Erythritol | a-methyl D glucosyl | Rhamnose | Cellobiose | Melezitose | a-methyl D manoside | Xylitol | ONPG | D-Arabinose | Citrate utilization | Malonate utilization | Sorbose |
| **BDP 1** | **-** | **+** | **-** | **+** | **-** | **+** | **+** | **-** | **+** | **+** | **+** | **+** | **-** | **-** | **-** | **-** | **+** | **-** | **+** | **-** | **+** | **-** | **-** | **-** | **+** | **+** | **-** | **+** | **-** | **-** | **-** | **+** | **-** | **-** | **-** | **-** | **+** | **+** | **-** | **-** |
| BDP 2 | **-** | **+** | **-** | **+** | **-** | **+** | **+** | **-** | **+** | **+** | **+** | **+** | **-** | **-** | **-** | **-** | **+** | **-** | **+** | **-** | **+** | **-** | **-** | **-** | **+** | **+** | **-** | **+** | **-** | **-** | **-** | **+** | **-** | **-** | **-** | **-** | **+** | **+** | **-** | **-** |
| BDP 3 | **-** | **+** | **-** | **+** | **-** | **+** | **+** | **-** | **+** | **+** | **+** | **+** | **-** | **-** | **-** | **-** | **+** | **-** | **+** | **-** | **+** | **-** | **-** | **-** | **+** | **+** | **-** | **+** | **-** | **-** | **-** | **+** | **-** | **-** | **-** | **-** | **+** | **+** | **-** | **-** |
| BDP 4 | **-** | **+** | **-** | **+** | **-** | **+** | **+** | **-** | **+** | **+** | **+** | **+** | **-** | **-** | **-** | **-** | **+** | **-** | **+** | **-** | **+** | **-** | **-** | **-** | **+** | **+** | **-** | **+** | **-** | **-** | **-** | **+** | **-** | **-** | **-** | **-** | **+** | **+** | **-** | **-** |
| **BDP 10** | **-** | **+** | **+** | **+** | **-** | **+** | **-** | **-** | **+** | **+** | **+** | **-** | **-** | **-** | **-** | **-** | **-** | **-** | **-** | **-** | **-** | **-** | **-** | **-** | **+** | **-** | **-** | **+** | **-** | **-** | **-** | **-** | **-** | **-** | **-** | **-** | **+** | **-** | **-** | **-** |
| BDP 11 | **+** | **+** | **+** | **+** | **-** | **+** | **-** | **-** | **-** | **-** | **+** | **-** | **-** | **-** | **-** | **-** | **-** | **-** | **-** | **-** | **-** | **-** | **-** | **-** | **-** | **-** | **-** | **-** | **+** | **-** | **+** | **-** | **-** | **-** | **-** | **-** | **+** | **+** | **+** | **-** |
| **BDP 12** | **-** | **+** | **+** | **+** | **-** | **+** | **-** | **-** | **-** | **-** | **+** | **+** | **-** | **-** | **-** | **+** | **-** | **+** | **-** | **-** | **+** | **-** | **-** | **-** | **+** | **+** | **-** | **-** | **+** | **-** | **-** | **+** | **+** | **-** | **-** | **+** | **+** | **+** | **-** | **+** |
| BDP 13 | **+** | **+** | **+** | **+** | **-** | **+** | **-** | **-** | **-** | **-** | **+** | **-** | **-** | **-** | **-** | **-** | **-** | **-** | **-** | **-** | **-** | **-** | **-** | **-** | **-** | **-** | **-** | **-** | **+** | **-** | **+** | **-** | **-** | **-** | **-** | **-** | **+** | **+** | **+** | **-** |
| BDP 15 | **+** | **+** | **+** | **+** | **-** | **+** | **-** | **-** | **-** | **-** | **+** | **-** | **-** | **-** | **-** | **-** | **-** | **-** | **-** | **-** | **-** | **-** | **-** | **-** | **-** | **-** | **-** | **-** | **+** | **-** | **+** | **-** | **-** | **-** | **-** | **-** | **+** | **+** | **+** | **-** |
| **BDP 18** | **-** | **+** | **+** | **+** | **-** | **+** | **-** | **-** | **-** | **-** | **+** | **-** | **-** | **-** | **-** | **-** | **-** | **-** | **-** | **-** | **-** | **-** | **-** | **-** | **-** | **-** | **-** | **-** | **-** | **-** | **-** | **-** | **-** | **-** | **-** | **-** | **+** | **+** | **-** | **-** |
| BDP 19 | **+** | **+** | **+** | **+** | **-** | **+** | **-** | **+** | **-** | **-** | **+** | **-** | **-** | **-** | **-** | **-** | **-** | **-** | **-** | **-** | **-** | **-** | **-** | **-** | **-** | **-** | **-** | **-** | **-** | **-** | **-** | **-** | **-** | **-** | **-** | **-** | **+** | **+** | **-** | **-** |
| **BDP 20** | **-** | **+** | **+** | **+** | **-** | **+** | **-** | **-** | **+** | **+** | **+** | **-** | **-** | **-** | **-** | **-** | **-** | **-** | **-** | **-** | **-** | **-** | **-** | **-** | **+** | **-** | **-** | **+** | **-** | **-** | **-** | **-** | **-** | **-** | **-** | **-** | **+** | **-** | **-** | **-** |
| BDP 21 | **-** | **+** | **+** | **+** | **-** | **+** | **-** | **-** | **+** | **+** | **+** | **-** | **-** | **-** | **-** | **-** | **-** | **-** | **-** | **-** | **-** | **-** | **-** | **-** | **+** | **-** | **-** | **+** | **-** | **-** | **-** | **-** | **-** | **-** | **-** | **-** | **+** | **-** | **-** | **-** |
| **BDP 23** | **-** | **+** | **+** | **+** | **-** | **+** | **-** | **-** | **-** | **-** | **+** | **-** | **-** | **-** | **-** | **-** | **-** | **-** | **-** | **-** | **-** | **-** | **-** | **-** | **-** | **-** | **-** | **-** | **-** | **-** | **-** | **-** | **-** | **-** | **-** | **-** | **+** | **+** | **-** | **-** |
| **BDP 24** | **-** | **+** | **+** | **+** | **-** | **+** | **-** | **-** | **+** | **-** | **+** | **-** | **-** | **-** | **-** | **-** | **-** | **-** | **-** | **+** | **-** | **-** | **-** | **-** | **-** | **-** | **-** | **-** | **-** | **-** | **-** | **+** | **-** | **-** | **-** | **-** | **+** | **-** | **-** | **-** |

+ indicates positive result/isolate is able to utilize the C source
- indicates negative result/ isolate is unable to utilize the C source

Table S2. The identity scores based on sequence homology for the 16S rRNA sequences from the BDP bacterial isolates

| **Isolate No.** | **Closest match** | **Fragment size** | **Total score** | **Query coverage** | **Accession no.** | **Maximum identity** | **GC %** | **Gaps** |
| --- | --- | --- | --- | --- | --- | --- | --- | --- |
| BDP1 | *Acinetobacter calcoaceticus* strain PVAS6 | 1443 | 2427 | 100% | GU130530 | 97% | 53.49 | 11 |
| BDP2 | *Acinetobacter lwoffii* strain KNA-3 | 1443 | 2555 | 99% | HM854251 | 99% | 53.04 | 2 |
| BDP3 | *Acinetobacter calcoaceticus* strain PVAS6 | 1443 | 2427 | 100% | GU130530 | 97% | 53.49 | 11 |
| BDP4 | *Acinetobacter calcoaceticus* strain PVAS6 | 1443 | 2427 | 100% | GU130530 | 97% | 53.49 | 11 |
| BDP10 | *Hydrogenophagaatypica* strain BSB41.8 | 1496 | 2760 | 100% | NR_029023 | 100% | 55.61 | 0 |
| BDP11 | *Leucobacter*sp. K540-B | 1390 | 2377 | 100% | NR_112961 | 98% | 55.82 | 4 |
| BDP12 | *Albidiferax* sp. 7A-189 | 1439 | 2510 | 99% | KF441628 | 98% | 54.55 | 3 |
| BDP13 | *Leucobactersalsicius*M1-8 | 1441 | 2662 | 100% | NR_117298 | 100% | 56.26 | 0 |
| BDP15 | *Leucobacterluti*strain RF6 | 1441 | 2521 | 99% | NR_042425 | 98% | 56.45 | 3 |
| BDP18 | *Leptothrix*sp. S1.1 | 1443 | 2519 | 99% | DQ241397 | 98% | 54.24 | 4 |
| BDP19 | *Zoogloearesiniphila* strain DhA-35 | 1418 | 2414 | 98% | NR_027188 | 98% | 55.18 | 3 |
| BDP20 | *Hydrogenophagabisanensis* strain K102 | 1435 | 2558 | 99% | NR_044268 | 99% | 55.95 | 0 |
| BDP21 | *Hydrogenophagaatypica* strain BSB41.8 | 1443 | 2741 | 99% | NR_029023 | 99% | 55.62 | 1 |
| BDP23 | *Zoogloearesiniphila*strain DhA-35 | 1414 | 2414 | 99% | NR_027188 | 98% | 55.20 | 2 |
| BDP24 | *Acidovoraxfacilis*CCUG 2123 | 1414 | 2414 | 99% | NR_024935 | 98% | 54.39 | 2 |

Max score was equal to total score
E value for all identity scores were 0.

Table S3. Sequence homology of *aioA* sequences

| **Isolate No.** | **Closest match** | **Fragment size** | **Total score** | **Query coverage** | **Accession No.** | **Maximum identity** | **Gaps** |
| --- | --- | --- | --- | --- | --- | --- | --- |
| BDP1 | *Acinetobacter lwoffii* | 353 | 640 | 86 % | AJF38909 | 99 % | 0 |
| BDP2 | Leptothrix sp. S1-1 | 352 | 700 | 100 % | AJF38913 | 94 % | 0 |
| BDP3 | *Acinetobacter lwoffii* | 353 | 639 | 86 % | AJF38909 | 99 % | 0 |
| BDP4 | *Acinetobacter lwoffii* | 353 | 639 | 86 % | AJF38909 | 99 % | 0 |
| BDP10 | *Hydrogenophagabisanensis* | 352 | 704 | 100 % | AJF38911 | 95 % | 0 |
| BDP12 | *Rhodoferaxferrireducens* | 352 | 734 | 100 % | WP011465357 | 99 % | 0 |
| BDP18 | *Leptothrix*sp. S1.1 | 352 | 731 | 100 % | ABY19320 | 99 % | 0 |
| BDP20 | *Hydrogenophagaatypica* | 352 | 704 | 100 % | AJF38910 | 95 % | 0 |
| BDP23 | *Pseudomonas* sp. 46 | 352 | 733 | 100 % | ABY19333 | 99 % | 0 |
| BDP24 | *Variovorax*sp. 4-2 | 352 | 660 | 100 % | ABY19319 | 87 % | 0 |

Max score was equal to total score
E value for all identity scores were 0.

Table S4. Total content of PLFA extracted from bacterial strains grown in different concentration of As(III).

| **Isolate name** | **Concentration of As(III) in growth medium** | **ΣPLFA**  **µg/g*** |
| --- | --- | --- |
| *Acinetobacter lwoffi*strain BDP2 | 1 mM | 21.91 |
|  | 2 mM | 14.09 |
|  | 5 mM | 17.02 |
|  | 10 mM | 16.73 |
| *Hydrogenophagaatypica*strain BDP10 | 1 mM | 53.02 |
|  | 2 mM | 9.46 |
|  | 5 mM | 67.59 |
|  | 10 mM | 56.39 |
| *Albidiferax*sp. strain BDP12 | 1 mM | 26.24 |
|  | 2 mM | 42.57 |
|  | 5 mM | 13.86 |
|  | 10 mM | 27.05 |
| *Leptothrix*sp. strain BDP18 | 1 mM | 22.44 |
|  | 2 mM | 14.43 |
|  | 5 mM | 19.58 |
|  | 10 mM | 14.25 |
| *Hydrogenophagabisanensis* strain BDP20 | 1 mM | 1007.6 |
|  | 2 mM | 104.95 |
|  | 5 mM | 44.34 |
|  | 10 mM | 336.87 |
| *Pseudomonas* sp. strain BDP23 | 1 mM | 672.15 |
|  | 2 mM | 35.35 |
|  | 5 mM | 41.99 |
|  | 10 mM | 85.12 |
| *Acidivoraxfacilis*strain BDP24 | 1 mM | 86.41 |
|  | 2 mM | 26.86 |
|  | 5 mM | 16.22 |
|  | 10 mM | 17.43 |

*Dry weight of cell pellet
